# Icariin Ameliorates Diabetic Cardiomyopathy Through Apelin/Sirt3 Signalling to Improve Mitochondrial Dysfunction

**DOI:** 10.3389/fphar.2020.00256

**Published:** 2020-03-19

**Authors:** Tingjuan Ni, Na Lin, Xingxiao Huang, Wenqiang Lu, Zhenzhu Sun, Jie Zhang, Hui Lin, Jufang Chi, Hangyuan Guo

**Affiliations:** ^1^Department of Cardiology, Zhejiang University School of Medicine, Hangzhou, China; ^2^Department of Cardiology, Zhejiang Chinese Medical University, Hangzhou, China; ^3^Department of Cardiology, The First Clinical Medical College, Wenzhou Medical University, Wenzhou, China; ^4^Department of Cardiology, Shaoxing people’s Hospital (Shaoxing hospital, Zhejiang University School of Medicine), Shaoxing, China

**Keywords:** icariin, diabetic cardiomyopathy, DCM, mitochondrial dysfunction, cardiac dysfunction, Apelin/Sirt3

## Abstract

Myocardial contractile dysfunction in diabetic cardiomyocytes is a significant promoter of heart failure. Herein, we investigated the effect of icariin, a flavonoid monomer isolated from *Epimedium*, on diabetic cardiomyopathy (DCM) and explored the mechanisms underlying its unique pharmacological cardioprotective functions. High glucose (HG) conditions were simulated *in vitro* using cardiomyocytes isolated from neonatal C57 mice, while DCM was stimulated *in vivo* in db/db mice. Mice and cardiomyocytes were treated with icariin, with or without overexpression or silencing of Apelin and Sirt3 *via* transfection with adenoviral vectors (Ad-RNA) and specific small hairpin RNAs (Ad-sh-RNA), respectively. Icariin markedly improved mitochondrial function both *in vivo* and *in vitro*, as evidenced by an increased level of mitochondrial-related proteins *via* western blot analysis (PGC-1α, Mfn2, and Cyt-b) and an increased mitochondrial membrane potential, as observed *via* JC-1 staining. Further, icariin treatment decreased cardiac fibrogenesis (Masson staining), and inhibited apoptosis (TUNEL staining). Together, these changes improved cardiac function, according to multiple transthoracic echocardiography parameters, including LVEF, LVSF, LVESD, and LVEDD. Moreover, icariin significantly activated Apelin and Sirt3, which were inhibited by HG and DCM. Importantly, when Ad-sh-Apelin and Ad-sh-Sirt3 were transfected in cardiomyocytes or injected into the heart of db/db mice, the cardioprotective effects of icariin were abolished and mitochondrial homeostasis was disrupted. Further, it was postulated that since Ad-Apelin induced different results following increased Sirt3 expression, icariin may have attenuated DCM development by preventing mitochondrial dysfunction through the Apelin/Sirt3 pathway. Hence, protection against mitochondrial dysfunction using icariin may prove to be a promising therapeutic strategy against DCM in diabetes.

## Introduction

In the present decade, the prevalence of diabetes and its associated complications have been steadily increasing, particularly within developing countries. In fact, diabetes has been reported by the World Health Organization as the disease requiring the most intervention; its microvascular and macrovascular complications account for the most common causes of death in patients with type 2 diabetes and heart failure (Kosiborod et al., 2018). Despite the introduction of antidiabetic drugs in the United States (US), deaths due to heart failure in diabetic patients have not declined from 1985-2015 (Cheng et al., 2018). Instead, diabetic cardiomyopathy (DCM) has become a proximate cause of heart failure among diabetic patients (Zamora and Villena, 2019), and is one of the primary causes for the reduced functioning of diabetic cardiomyocytes (Dillmann, 2019). To date, however, the mechanism of DCM remains uncharacterized.

Mitochondria are critical organelles for energy production *via* oxidative phosphorylation, a reaction that is conjugated with the production of reactive oxygen species (ROS), which either induces diverse molecular signals or cell persecution and death in cardiomyocytes (Zhou and Tian, 2018). Recent studies have revealed that high glucose (HG) concentrations induce a loss of mitochondrial networks and increased reactive ROS in cardiomyocytes (Evangelista et al., 2019). In addition, increasing evidence demonstrates that mitochondrial dysfunction contributes to the pathogenesis of DCM (Schilling, 2015; Verma et al., 2017; Zhou and Tian, 2018; Mahalakshmi and Kurian, 2019). Alternatively, the overexpression of the mitochondrial protein Mfn2 restores mitochondrial dysfunction and prevents the development of DCM (Hu et al., 2019). The mitochondrion-targeted methylglyoxal- sequestering compound MitoGamide exhibits cardioprotective effects in an experimental model of DCM (Tate et al., 2019), thereby suggesting that the alleviation of mitochondrial dysfunction may provide a novel therapeutic approach for reversing the development of DCM.

Icariin (C_33_H_40_O_15_, ICA) is a flavonoid monomer isolated from the herb *Epimedium*, and it has garnered attention for its prospects in pharmacology, including its cardioprotective functions (Zhou et al., 2014; Meng et al., 2015). Icariin protects cardiomyocytes against oxidative stress by activating sirtuin-1 (Wu et al., 2018), thereby alleviating oxidative stress-induced cardiac apoptosis *via* mitochondrial protection (Song et al., 2016), and protects cardiomyocytes from apoptosis induced by hypertrophy *via* inhibition of ROS-dependent JNK (Zhou et al., 2014). However, the protective effect of icariin against DCM has not yet been reported.

Similarly, Apelin contains a G-protein-coupled receptor, APJ, and exhibits positive cardiovascular effects (Japp et al., 2010). Apelin is a gene composed of 77 amino acid proproteins that are readily cleaved into apelin-36, apelin-17, and apelin-13, which are bioactive Apelin fragments (Bertrand et al., 2015). In particular, apelin-13 is a predominant isoform of Apelin found in cardiac tissue (Serpooshan et al., 2015). Apelin is also able to mitigate HG-induced cardiac gap dysfunctions in cardiomyocytes *via* the AMPK pathway (Li et al., 2018). A related study has shown that Apelin gene therapy alleviates DCM through VEGF to increase myocardial vascular density *via* Sirt3 upregulation (Zeng et al., 2014). Sirt3 is a nicotinamide adenine dinucleotide (NAD+)-dependent deacetylase that is highly conserved and localized in the mitochondrial matrix (Hirschey et al., 2010; Heinonen et al., 2019). Further, Sirt3 activates PRDX3 to attenuate mitochondrial oxidative injuries and apoptosis induced by ischaemia-reperfusion injury (Wang et al., 2020). Hence, the upregulation of Sirt3 induced by Apelin gene therapy has been shown to prevent heart failure by increasing autophagy in patients with diabetes (Hou et al., 2015). However, the role Apelin and Sirt3 take in protecting against DCM *via* reducing mitochondrial dysfunction has not yet been elucidated.

In the present study, we sought to define the protective effect of icariin on DCM, while elucidating its underlying mechanism. We hypothesized that icariin would effectively alleviate mitochondrial dysfunction in cardiomyocytes during DCM through Apelin/Sirt3 signalling.

## Materials and Methods

### Reagents

Icariin (purity 98.75%) and JC-1 dye were purchased from MedChem Express (MCE, NJ, USA). The dihydroethidium (DHE) probe and mitochondria isolation kits were obtained from Beyotime Biotechnology (Jiangsu, China). The *in situ* cell death detection kit (Roche) was obtained from Sigma-Aldrich (St. Louis, MO, USA); and the primary antibodies against Sirt3, Sirt1, PGC-1α, Mfn2, Cyt-b, and β-actin, as well as the goat anti-rabbit and goat anti-mouse secondary antibodies were obtained from Abcam Biotechnology (Cambridge, MA, USA). The primary antibody against Apelin was obtained from Signalway Antibody (SAB, USA). Empty adenoviral vectors (Ad-EV) and recombinant adenoviral vectors expressing Apelin (Ad-Apelin), Sirt3 (Ad-Sirt3) or Apelin-specific small hairpin RNA (Ad-sh-Apelin), as well as Sirt3-specific small hairpin RNA (Ad-sh-Sirt3), were purchased from Hanbio Technology Ltd. (Shanghai, China). The titer of the adenoviruses was approximately 1.2×10^10^ PFU/mL.

### Animal Experiments

Male db/db mice and db/+ mice (7 weeks old) were obtained from the Model Animal Research Center of Nanjing University (Nanjing, China). All animals received humanitarian care in adherence with the Institutes of Shaoxing People's Hospital Health Guidelines on the Use of Laboratory Animals. Both db/+ mice and db/db mice were fed a normal diet for four weeks. Thereafter, the adenovirus was injected into their myocardium. Mice were anesthetized with 2.5% isoflurane, and maintained under this condition for the duration of the operation. After the heart was exposed, the adenovirus (using a 50μL needle, Hamilton, 705RN, USA) was injected into the left ventricle free wall (10 μL for each of four sites). Mice (every group n = 10) were then treated with or without the icariin (30mg/kg) for an additional 16 weeks.

### Transthoracic Echocardiography Recordings

A Philips iE33 system (Philips Medical, Best, Netherlands) equipped with an s5-1 probe (12-14 MHz) was used to measure the left ventricular end-diastolic diameter (LVEDD) and left ventricular end-systolic diameter (LVESD). A computer algorithm was used to assess the left ventricular ejection fraction (LVEF) and left ventricular fraction shortening (LVFS).

### Transmission Electron Microscopy

The left ventricle myocardial tissues were fixed, dehydrated stepwise in an alcohol series, embedded, sectioned into 50-60 mm slices with an LKB-1 ultramicrotome, and double stained with 3% uranyl acetate lead citrate. The morphological mitochondrion changes in the myocardium were visualized with an electron microscope (JEM-2000EX TEM, Tokyo, Japan).

### Histological Analysis

The left ventricular myocardial tissues were embedded in paraffin, and sectioned (5-mm thickness). Masson staining was then implemented according to standard procedures to assess cardiac collagen content.

### Primary Culture of Neonatal Cardiomyocytes

Primary cardiomyocyte cultures were obtained from the ventricles of newborn (1-day-old) C57 mice according to a previously published protocol (Sreejit et al., 2008). The cardiomyocytes were incubated in a low-glucose medium (5.5 mmol/L glucose) or HG medium (25 mmol/L glucose) and treated with or without 7.5μM, 15μM, or 30μM icariin for 12, 24 or 48 h. Cardiomyocytes were transfected with the adenovirus (10 μL/mL, MOI: 100:1) in serum-free Dulbecco's Modified Eagle Medium (DMEM) for 6-8 h. The cells were then treated with HG or normal medium with or without icariin for an additional 24 h.

### Determination of Apoptosis and ROS Production in Cardiac Tissue and Cardiomyocytes

Apoptosis in the myocardial tissue and cardiomyocytes was determined by TUNEL staining using left ventricular myocardial tissue embedded in paraffin according to the manufacture's protocol. Dihydroethidium (DHE) staining was used to detect intracellular levels of superoxide anions in frozen sections of the tissues.

### Mitochondrial Membrane Potential Measurement

JC-1 staining and Mito-tracker Red CMXRos staining were used to measure the mitochondrial membrane potential. Cardiomyocytes were stained with 2.5 mmol/L JC-1 dye for 30 min at 37°C in the dark. The images were obtained using fluorescence microscopy (400 ×).

### Mitochondria Isolation

Mitochondria were isolated from fresh cardiac tissues and cardiomyocytes using the Mitochondria Isolation Kit (Beyotime, Jiangsu, China) according to the manufacturer's instructions.

### Western Blotting Analysis

Protein samples were obtained from cardiac tissues and cardiomyocytes, and their mitochondria were denatured *via* boiling. Western blotting was carried out according to a previously described protocol (Ni et al., 2019). The primary antibodies used were Sirt3, Sirt1, Apelin, Mfn2, PGC-1α, Cyt-b and β-actin.

### Statistical Analysis

All experiments were repeated in triplicate. All data are presented as mean ± standard deviation. Multiple groups were compared using one-way ANOVA and the results were qualified by Bonferroni following the event. Unpaired Student's *t* tests were performed on the two groups. P values < 0.05 were considered to be statistically significant.

## Results

### Icariin Alleviated Cardiac Dysfunction and Rescued Mitochondrial Dysfunction in db/db Mouse Hearts

**Figure 1:**
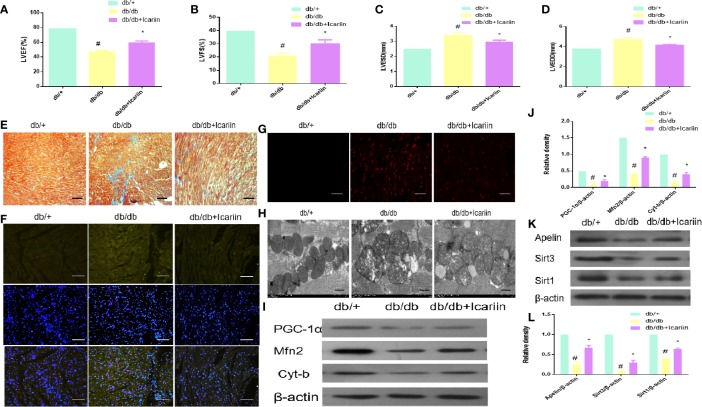
Icariin improves the cardiac function in the diabetic heart, improves mitochondrial dysfunction in diabetic myocardium and increases Apelin expression. Db/db mice (n = 10) were treated with icariin (30 mg/kg), db/+mice (n = 10). **(A)** LVEF (%) recordings; **(B)** LVFS (%) recordings; **(C)** LVESD (mm) recordings; **(D)** LVEDD (mm) recordings; **(E)**, Collagen deposition measured by Masson staining (blue indicates collagen deposition); **(F)** Apoptotic rate of cardiomyocytes measured by TUNEL staining; **(G)** ROS production measured by DHE staining; **(H)**, Mitochondrial morphology measured by TEM; **(I)** Protein expression with representative gel blots of PGC‐1α, Mfn2, Cyt-b, and β-actin (loading control); **(J)** Relative levels of PGC‐1α, Mfn2, Cyt-b; **(K)**, Protein expression with representative gel blots of Apelin, Sirt1 and Sirt3; **(L)** Relative levels of Apelin, Sirt1 and Sirt3. ^#^P < 0.05 vs db/+and *P < 0.05 vs db/db; experiments were performed in triplicate.

Icariin significantly increased cardiac function performance, resulting in increased LVEF and LVFS, and decreased LVEDD and LVESD in db/db mice. (**Figures 1A–D**, P < 0.05). As demonstrated by Masson staining, collagen deposition was reduced in the myocardium of icariin-treated db/db mice (**Figure 1E**). On the other hand, TUNEL staining revealed that icariin treatment rescued the apoptotic rate of cardiomyocytes (**Figure 1F**). Moreover, the myocardial tissue of db/db mice showed a greater production of ROS, which was alleviated by icariin, as shown by DHE staining (**Figure 1G**). TEM revealed that in DCM, the fibrous region of mitochondria had an uneven, more fragmented, and swollen appearance, causing them to lose their recognizable crest; however, these characteristics were significantly mitigated in icariin-treated mice (**Figure 1H**). Further, the mitochondrial dynamics-related protein, Mfn2, mitochondrial biogenesis regulation protein, PGC‐1α, and mitochondrial gene, cytochrome-b (Cyt-b), were upregulated in the icariin-treated db/db mice (**Figures 1I, J**, P < 0.05). We also observed a downregulation in the expression of the cardiac myocardium gene Apelin and the mitochondrial matrix gene Sirt3 in the hearts of db/db mice following DCM, but their expression increased following icariin treatment. As previously reported, Sirt1 expression was consistent with this change (Saito et al., 2016; Ding et al., 2018) (**Figures 1K, L**, P < 0.05). These results indicated the amelioration of excessive mitochondrial damage in DCM following icariin treatment, which exerted cardioprotective effects by reversing mitochondrial dysfunction in DCM and may thus mediate Apelin to exert its positive effect.

### Icariin Rescued the Impaired Mitochondria and Reduced Apoptosis in HG-Treated Cardiomyocytes

**Figure 2:**
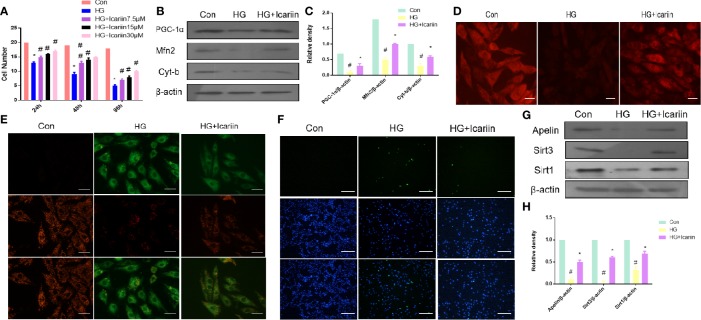
Icariin rescues impaired mitochondria, reduces apoptosis in cardiomyocytes, and increases Apelin expression. **(A)** Cardiomyocytes were treated with 7.5, 15, or 30 µM icariin for 24, 48, and 96 h, followed by DNA quantification to determine cell number/cell proliferation, after treatment with or without 25 mmol/L HG. Cardiomyocytes were then incubated with or without icariin (30μM) under HG (25mmol/L) stimulation. **(B)** Protein expression with representative gel blots of PGC‐1α, Mfn2, Cyt-b, and β-actin (loading control); **(C)** Relative levels of PGC‐1α, Mfn2, and Cyt-b. **(D)** Mitochondrial membrane potential measured by MitoTracker Red staining; **(E)** Mitochondrial membrane potential measured by JC-1 staining. Magnification ×400; **(F)** Apoptotic rate of cardiomyocytes measured by TUNEL staining. **(G)** Protein expression with representative gel blots of Apelin, Sirt1 and Sirt3, and β-actin (loading control); **(H)** Relative levels of Apelin, Sirt1 and Sirt3. ^#^P < 0.05 vs Con and *P < 0.05 vs HG; experiments were performed in triplicate.

We next sought to further confirm that mitochondrial dysfunction is required for DCM in hyperglycemia. Firstly, we observed that the number of HG-treated cardiomyocytes was significantly higher following incubation with icariin for 12h, 24h, or 48 h at concentrations of 7.5, 15, or 30 µM, compared to the HG group. For all subsequent experiments, icariin was used at a concentration of 30 µM (**Figure 2A**, P < 0.05). Secondly, we found that the expression of mitochondrion-related proteins, including PGC‐1α, Mfn2, and Cyt-b, decreased in HG- treated cardiomyocytes, however, the expression of these proteins significantly increased in the HG+ icariin group (**Figures 2B, C**, P < 0.05). As shown in **Figure 2E**, icariin increased the mitochondrial membrane potential, as revealed by the transition from red fluorescence to green fluorescence *via* JC-1 staining, Meanwhile, staining the mitochondria with MitoTracker Red (**Figure 2D**) revealed that the loss of mitochondrial membrane potential significantly reduced red fluorescence in the HG group, which was reversed following treatment with icariin. Next, as expected, the level of apoptosis in cardiomyocytes within the icariin + HG group was significantly reduced relative to that of the HG group (**Figure 2F**). Similar to the results obtained in the heart of db/db mice, a marked decrease in the expression of Apelin and Sirt3 was observed in the HG group (**Figures 2G, H**, P < 0.05), while icariin treatment dramatically alleviated the reduction in Apelin and Sirt3 expression, as previously reported, Sirt1 expression was consistent with this change (Saito and Asai, 2016; Ding and Feng, 2018). Cumulatively, these results confirm that *in vitro* icariin prevents HG-induced mitochondrial dysfunction-mediated cardiac apoptosis and may regulate the expression of Apelin and Sirt3.

### Apelin Inhibition Abolished the Cardioprotective Effect of Icariin on Mitochondrial Dysfunction in HG-Treated Cardiomyocytes and Diabetic Mice

**Figure 3:**
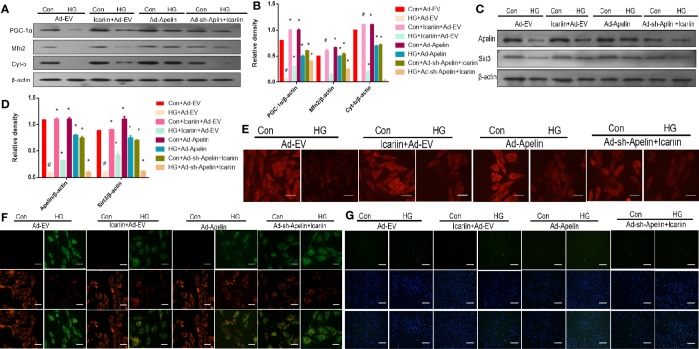
Icariin fails to exert protective effects in cardiomyocytes transfected with Ad-sh-Apelin. Cardiomyocytes were transfected with Ad-EV, Ad-Apelin, and Ad-sh-Apelin (10 μL/mL, MOI: 100:1) for 6-8 hours, and then incubated with or without icariin (30μM) under HG (25mmol/L) stimulation. **(A)** Protein expression with representative gel blots of Apelin, Sirt3 (loading control); **(B)** Relative levels of Apelin and Sirt3; **(C)** Protein expression with representative gel blots of PGC‐1α, Mfn2, Cyt-b, and β-actin (loading control); **(D)** Relative levels of PGC‐1α, Mfn2 and Cyt-b. **(E)** Mitochondrial membrane potential measured by MitoTracker Red staining. **(F)** Mitochondrial membrane potential measured by JC-1 staining. **(G)** Apoptotic rate of cardiomyocytes measured by TUNEL assay. ^#^P < 0.05 vs Con+Ad-EV and *P < 0.05 vs HG+Ad-EV, experiments were performed in triplicate.

We next explored the molecular mechanisms underlying the cardioprotective effects of icariin in HG-treated cardiomyocytes, while also deciphering whether Apelin and Sirt3 are integral components for the cardioprotective effects of icariin. As shown in **Figures 3A, B** (P < 0.05), in HG-treated cardiomyocytes without the overexpression of Apelin icariin treatment caused a significant increase in the expression of PGC‐1α, Mfn2, and Cyt-b, compared to the HG + Ad-EV group. Moreover, the HG + Ad-Apelin group evidently expressed mitochondrion-related proteins at levels comparable to, or greater than, that of the HG + icariin group. Alternatively, Ad-sh-Apelin administration significantly reversed the effect of icariin, thereby contributing to the offsetting of its cardioprotective function, as demonstrated by a decrease in mitochondrial membrane potential, as detected *via* MitoTracker Red staining (**Figure 3E**) and JC-1 staining (**Figure 3F**). Moreover, cardiomyocyte apoptosis was increased in the HG + icariin + Ad-sh-Apelin group compared to the HG + icariin + Ad-EV group (**Figure 3G**). Interestingly, Apelin significantly directed the expression of Sirt3 in Apelin-overexpressing HG-treated cardiomyocytes and the HG + Ad-sh-Apelin + icariin group (**Figures 3C, D**, P < 0.05).

**Figure 4:**
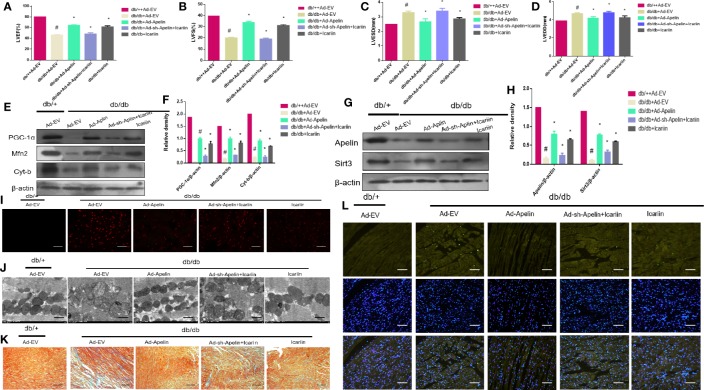
Icariin fails to exert protective effects in diabetic mice injected with Ad-sh-Apelin. Db/+mice (n = 10) and db/db mice (n = 10) fed with or without icariin (30 mg/kg) were injected with Ad-EV, Ad-Apelin, Ad-sh-Apelin (10 μL at each of four sites, 1.2×10^10^ PFU/mL) into the left ventricle free wall. **(A)** LVEF (%) recordings; **(B)** LVFS (%) recordings; **(C)** LVESD (mm) recordings; **(D)** LVEDD (mm) recordings; **(E)** Protein expression with representative gel blots of PGC‐1α, Mfn2, Cyt-b, and β-actin (loading control); **(F)** Relative levels of PGC‐1α, Mfn2 and Cyt-b; **(G)** Protein expression with representative gel blots of Apelin, Sirt3 and β-actin (loading control); **(H)** Relative levels of Apelin and Sirt3. **(I)** ROS production measured by DHE staining; **(J)** Mitochondrial morphology measured by TEM; **(K)** Collagen deposition measured by Masson staining (blue indicates collagen deposition). **(L)** Apoptotic rate of cardiomyocytes measured by TUNEL staining. ^#^P < 0.05 vs db/++Ad-EV and *P < 0.05 vs db/db+Ad-EV; experiments were performed in triplicate.

Subsequently, we explored whether icariin administration could restore DCM without overexpression of Apelin in diabetic hearts. As shown in **Figures 4A, D** (P < 0.05), Apelin inhibition counteracted the cardioprotective effects of icariin shown by a significant decrease in LVEF and LVFS and an evident increase in LVEDD and LVESD, compared to the HG + icariin + Ad-EV group. Furthermore, the alleviation of mitochondrial dysfunction in DCM by icariin was eliminated following an injection of Ad-sh-Apelin to db/db mice, as indicated by a greater decrease in mitochondrial protein expression (PGC‐1α, Mfn2, and Cyt-b) (**Figures 4E, F** P < 0.05), and increased production of ROS (**Figure 4I**). The mitochondria were also characterized by a greater loss of cristae, as well as increased swelling and significant distortion (**Figure 4J**). Moreover, increased mitochondrial dysfunction resulted in a greater level of collagen deposition in the heart tissue (**Figure 4K**) and increased cardiomyocyte apoptosis (**Figure 4L**). Although the expression of Sirt3 and Apelin in db/db mice injected with Ad-sh-Apelin was less than that in the db/db + icariin group (**Figures 4G, H**, P < 0.05), when Apelin was silenced in db/db mice or cardiomyocytes, icariin treatment failed to improve mitochondrial dysfunction and cardiac dysfunction.

### Apelin/Sirt3 signaling Is Involved in Icariin-Mediated Mitigation of Mitochondrial and Cardiac Dysfunction

**Figure 5:**
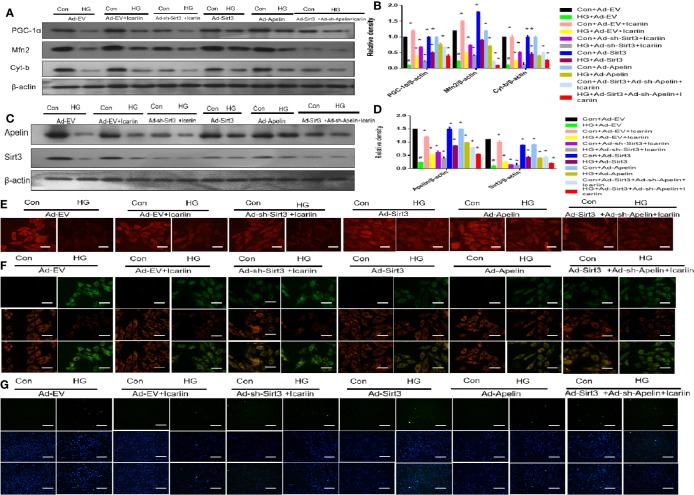
Icariin mitigates mitochondrial dysfunction through the Apelin/Sirt3 pathway. Cardiomyocytes were transfected with Ad-EV, Ad-Apelin, Ad-sh-Apelin, Ad-sh-Sirt3, or Ad-Sirt3 (10 μL/mL, MOI: 100:1) for 6-8 hours, and incubated with or without icariin (30 μM) under HG (25 mmol/L) stimulation. **(A)** Protein expression with representative gel blots of PGC‐1α, Mfn2, Cyt-b and β-actin (loading control); **(B)** Relative levels of PGC‐1α, Mfn2 and Cyt-b; **(C)** Protein expression with representative gel blots of Apelin, Sirt3, β-actin (loading control); **(D)** Relative levels of Apelin and Sirt3; **(E)** Mitochondrial membrane potential measured by MitoTracker Red staining. **(F)** Mitochondrial membrane potential measured by JC-1 staining; **(G)** Apoptotic rate of cardiomyocytes measured by TUNEL assay. ^#^P < 0.05 vs Con+Ad-EV and *P < 0.05 vs HG+Ad-EV; experiments were performed in triplicate.

Our previous results demonstrated that icariin alleviated DCM by increasing the expression of Apelin. In addition, we found that the expression level of Sirt3, a gene located in the mitochondrial matrix, changed with the expression of Apelin. Hence, we next explored the role of Sirt3 in the cardioprotective effect of icariin in HG-treated cardiomyocytes. As shown in **Figures 5A, B** (P < 0.05), although Sirt3 overexpression increased the expression of the mitochondrial proteins PGC‐1α, Mfn2, and Cyt-b, a significant decrease occurred when Apelin expression was silenced in HG-treated cardiomyocytes (**Figures 5C, D**, P < 0.05). This was also demonstrated by the decrease in mitochondrial membrane potential, as assessed by MitoTracker Red staining (**Figure 5E**) and JC-1 staining (**Figure 5F**), as well as the increase in apoptosis of cardiomyocytes (**Figure 5G**).

**Figure 6:**
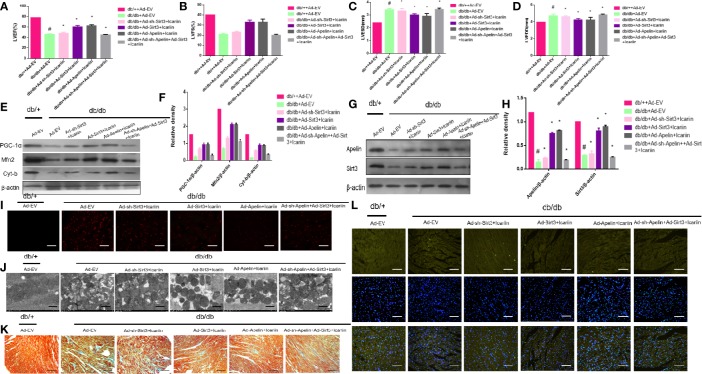
Icariin mitigates adverse cardiac dysfunction through the Apelin/Sirt3 signalling pathway. Db/+mice (n = 10) and db/db mice (n = 10) were injected with Ad-EV, Ad-Apelin, Ad-sh-Apelin,Ad-Sirt3 or Ad-sh-Sirt3 (10 μL at each of four sites, 1.2 × 10^10^ PFU/mL) into the left ventricle free wall, and treated with or without icariin (30 mg/kg). **(A)** LVEF (%) recordings; **(B)** LVFS (%) recordings; **(C)** LVESD (mm) recordings; **(D)** LVEDD (mm) recordings; **(E)** Protein expression with representative gel blots of PGC‐1α, Mfn2, Cyt-b and β-actin(loading control); **(F)** Relative levels of PGC‐1α, Mfn2, and Cyt-b; **(G)** Protein expression with representative gel blots of Apelin, Sirt3 and β-actin (loading control); **(H)** Relative levels of Apelin and Sirt3. **(I)** ROS production measured by DHE staining; **(J)** Mitochondrial morphology measured by TEM; **(K)** Collagen deposition measured by Masson staining (blue indicate collagen deposition). **(L)** Apoptotic rate of cardiomyocytes measured by TUNEL staining. ^#^P < 0.05 vs db/++Ad-EV and *P < 0.05 vs db/db+Ad-EV; experiments were performed in triplicate.

To further elucidate the role of Sirt3 in the mitochondrion-protective role against DCM following icariin treatment, the hearts of db/db mice were injected with Ad-sh-Sirt3, Ad-Sirt3, Ad-Apelin, or Ad-sh-Apelin. Consistent with previous results, Sirt3 elicited a marked effect when Apelin was not silenced. However, in the db/db + Ad-sh-Apelin + Ad-Sirt3 +icariin group, a significant decrease in LVEF and LVFS (**Figures 6A, B**, P < 0.05) as well as an evident increase in LVEDD and LVESD (**Figures 6C, D**, P < 0.05) were found relative to the results in the db/db + Ad-Sirt3 + icariin group. In addition, a lower expression of the mitochondrial proteins (**Figures 6E, F**, P < 0.05) and greater ROS production were revealed *via* DHE staining (**Figure 6I**). Additionally, the interfibrillar mitochondria displayed less uniformity and more fragmentation as well as a swollen appearance, as seen in the TEM analysis (**Figure 6J**). Lastly, increased apoptosis of the cardiomyocytes was demonstrated *via* TUNEL staining (**Figure 6L**), resulting in more collagen deposition in myocardial tissue, as measured by Masson staining (**Figure 6K**), due to adverse cardiac dysfunction. As expected, Sirt3 expression decreased when Ad-Sirt3 was transfected into the cardiomyocytes under the prerequisite of silent Apelin expression (**Figures 6G, H**, P < 0.05). Taken together, these results suggested that Apelin/Sirt3 signaling is directly responsible for the cardioprotective effects of icariin in DCM and under hyperglycemic conditions.

## Discussion

DCM is characterized by cardiac structural and functional abnormalities in diabetic patients without ischaemic or hypertensive heart disease (Jia et al., 2018). The researchers from the Framingham Heart Disease study were the first to report that the risk of heart failure in type 2 diabetes increases by 2-8 times, with 19% of individuals presenting symptoms of heart failure (Kannel et al., 1974). According to the latest report, the economic costs of diabetes and its complications account for an extensive global burden, especially in developing countries (Seuring et al., 2015). However, to date, no specific therapy or western medicine strategy has been shown to cure or alleviate DCM. In addition, a clear molecular mechanism for DCM has not been defined. In the present study, we systematically demonstrated that the cardioprotective effect of icariin against DCM relies on preventing mitochondrial dysfunction through the activation of Apelin/Sirt3 signaling.

Several previous studies have focused on mitochondria as a promising target for the treatment of DCM as mitochondrial dysfunction has been found to be associated with cardiovascular abnormalities in diabetes, which leads to DCM (Ni et al., 2016; Fan et al., 2017; Evangelista et al., 2019; Tang et al., 2019). In our study, mitochondrial dysfunction was apparent in the HG-treated cardiomyocytes and DCM, as demonstrated by the downregulated expression of the mitochondrial dynamic-related protein Mfn2, mitochondrial biogenesis regulation protein PGC‐1α, and mitochondrial gene Cyt-b. This was also revealed by the increased production of ROS from the mitochondria as determined by DHE staining, and by the loss of uniform appearance and development of more fragmented swelling within interfibrillar mitochondria, loss of discernible ridges as assessed by TEM, and decreased mitochondrial membrane potential as assessed by JC-1 staining and MitoTracker Red staining (**Figures 1H–J**, **2A–D**). Moreover, the expression of Apelin and Sirt3 was downregulated in HG-treated cardiomyocytes and DCM. Mounting evidence suggests that Apelin acts as an important adipokine against diabetes (Castan-Laurell et al., 2011; Czarzasta et al., 2019). Hence, as expected, the overexpression of Apelin reversed the mitochondrial dysfunction (**Figures 3A–F**). Our findings suggest that the endogenous activation of Apelin may be a potential therapeutic target for DCM.

Many studies have demonstrated that icariin, a polyphenol flavonoid (Meng et al., 2015), protects cardiomyocytes from injury and, therefore, elicits cardioprotective effects (Ke et al., 2015; Zheng et al., 2019). Interestingly, we found that icariin restored the cardiac function in DCM, as demonstrated by the decrease in LVEF and LVFS (**Figures 1A, B**) and the evident increase in LVEDD and LVESD (**Figures 1C, D**). Additionally, icariin alleviated mitochondrial dysfunction in HG-treated cardiomyocytes and the myocardium of db/db mice based on the expression of mitochondrial-related proteins, PGC‐1α, Mfn2 and Cyt-b, demonstrated with JC-1 staining, DHE staining, and TEM. (**Figures 1E–I**, **2A–D**). Surprisingly, icariin treatment was found to reduce the expression of Apelin and Sirt3 in HG-treated cardiomyocytes and db/db mice myocardium. (**Figures 1K, L**, **2F, G**).

**Figure 7:**
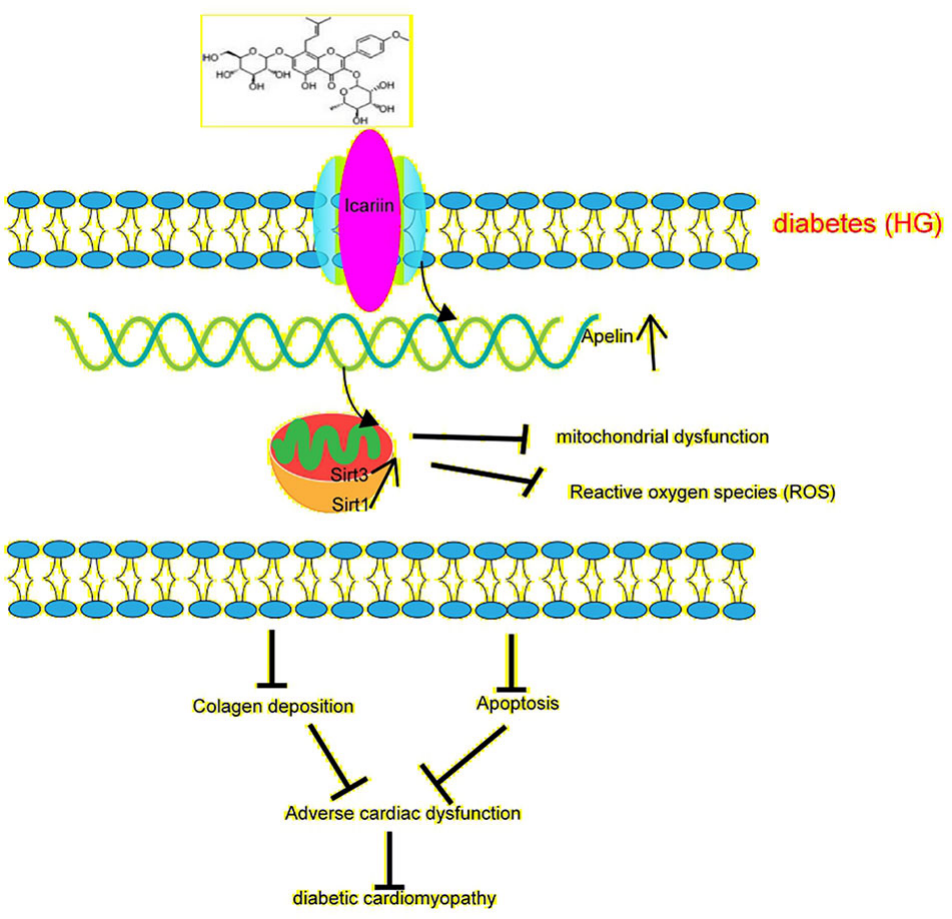
Schematic illustrating icariin's protection against diabetes-induced mitochondrial dysfunction in cardiomyocytes and cardiac dysfunction through Apelin/Sirt3 signaling. represents enhancement represents inhibition.

Emerging studies suggest that sirtuins play a critical role in diabetes (Turkmen, 2014). Sirtuins contain Sirt3 in the mitochondria (Javadipour et al., 2019), which is widely expressed in the heart. Moreover, Sirt3 has been found to prevent the development of DCM in the heart (Sun et al., 2018). In this study, we found that the overexpression of Sirt3 in HG-treated cardiomyocytes alleviated mitochondrial dysfunction (**Figures 5A, B, E, F**), compared to that achieved in the HG + Ad-EV group. More importantly, icariin increased the expression of Sirt3 (**Figures 2F, G**, **3K, L**), however, it did not affect the activity of Sirt3 in Ad-sh-Apelin transfected samples, HG-treated cardiomyocytes (**Figures 3C, D**) or in db/db mice injected with Ad-sh-Apelin (**Figures 4G, H**). Despite the overexpression of Sirt3, icariin had no cardioprotective effect in the presence of Apelin inhibition (**Figures 5A–G**, **6A–L**). Such findings strongly suggest that Apelin/Sirt3 signaling mediates the cardioprotective effects of icariin against the development of DCM (**Figure 7**).

## Conclusions

In the present study, we revealed that icariin can alleviate mitochondrial dysfunction and decrease apoptosis and adverse cardiac dysfunction in DCM. In addition, we found that its mechanisms are associated with Apelin/Sirt3 signaling. Together, the findings of this study demonstrate that icariin may serve as a promising therapeutic agent for the treatment and prevention of DCM.

## Data Availability Statement

All datasets generated for this study are included in the article/supplementary material.

## Ethics Statement

The animal study was reviewed and approved by Shaoxing People's Hospital Health Guidelines on the Use of Laboratory Animals.

## Author Contributions

Conceptualization: JC and HG. Methodology: TN. Software: NL. Validation: TN, HG, JC. Formal analysis: XH. Investigation: WL. Resources: ZS. Data curation: JZ. Writing—original draft preparation: TN. Writing—review and editing: NL. Visualization: HL. Supervision: XH. Project administration: JC. Funding acquisition: HG.

## Funding

This study was funded by the National Natural Science Foundation of China (81873120) and the Social Development Project of Public Welfare Technology Application in Zhejiang Province (LGF19H020006) and Shaoxing Science and Technology Project (LGD19H110002).

## Conflict of Interest

The authors declare that the research was conducted in the absence of any commercial or financial relationships that could be construed as a potential conflict of interest.

## Abbreviations

DCM, Diabetic cardiomyopathy; ROS, reactive oxygen species; Cyt-b, cytochrome-b; DHE, dihydroethidium; Ad-EV, Empty adenoviral vectors RNA; Ad-Apelin, recombinant adenoviral vector expressing Apelin; Ad-Sirt3, recombinant adenoviral vector expressing Sirt3; Ad-sh Apelin, recombinant adenoviral vector expressing Apelin-specific small hairpin RNA; Ad-sh-Sirt3, recombinant adenoviral vector expressing Sirt3-specific small hairpin RNA; LVEDD, left ventricular end-diastolic diameter; LVESD, left ventricular end-systolic diameter; LVEF, left ventricular ejection fraction; LVFS, left ventricular fraction shortening; HG, high glucose.
